# Lorazepam use during clinical trials of adults with bipolar mania episodes

**DOI:** 10.1016/j.conctc.2023.101122

**Published:** 2023-04-07

**Authors:** Daniel J. Safer

**Affiliations:** Departments of Psychiatry and Pediatrics, Johns Hopkins University School of Medicine, Baltimore, MD, 21087, USA

**Keywords:** Lorazepam, Mania, Bipolar, Antimanic, Placebo, Trials

## Abstract

**Background:**

Lorazepam has commonly been prescribed to reduce agitation during bipolar 1 mania trials. Its use has varied considerably by trial methodology and in clinical practice.

**Methods:**

The extent and amount of lorazepam treatment was recorded and analyzed from available brief, controlled trials of acute bipolar mania and in clinical reports in adults.

**Results:**

In 3-week, placebo-controlled clinical trials (n = 19), most manic subjects (79%) were treated with lorazepam to reduce agitation. This treatment was most prominent during the antimanic drug wash-out phase that preceded placebo-controlled trials. Doses of lorazepam administered during the first 7–10 days of the pre-trial and the early trial phases averaged 2.2 mg/day. These doses were one-third the lorazepam/clonazepam doses administered during placebo-controlled, non-washout trials. Far higher benzodiazepine doses for manic agitation were noted in emergency department reports. Intake enrollment was strikingly restricted only in placebo-controlled trials that used pretrial drug wash-out.

**Conclusions:**

Medication treatment conclusions from placebo-controlled, drug washout trials are not representative of clinical treatment for acute bipolar mania.

## 1 Introduction

A few months ago, I received a telephone call from a patient's wife reporting that her husband was in the midst of an acute manic episode, his fourth. He hadn't slept much for days; he was generally uncooperative and delusional; and his wife didn't have the evidence to clearly support an involuntary admission. Also, the patient was extremely evasive. I suggested to his wife that she increase his olanzapine. The next day she reported that he still slept very little and that his mental status was essentially unchanged. I then called in a prescription for clonazepam and advised that he be given 2 or more mg at night—along with 20 mg of olanzapine. He agreed to take two mg clonazepam and slept much better that night. She called daily for the next week and reported that her husband was now sleeping adequately and was slowly on the mend.

Afterward, I looked up studies on benzodiazepines for mania and decided to review this literature. Trial data on the subject of benzodiazepine treatment for mania are limited. Fortunately, there was a comprehensive, recent listing of controlled clinical trials of adults with bipolar manic disorder [1], and that list became the basis for my medication trial analyses.

In my review, I found that there are major limitations to drawing conclusions from placebo-controlled drug trials for adults with bipolar mania. First, most of the controlled trials are placebo-controlled which begin with a brief (1–3 days) pre-trial drug wash-out. The drug withdrawal process involves subjects rapidly being taken off their pre-trial antimanic medication (e.g., antipsychotics, lithium) before the actual trial, a procedure that has medical and psychiatric risks. Second, to limit the risks, trial enrollment has been very restrictive and selective. Excluded from those trials are the majority of prospective trial subjects including those with comorbidities (e.g., substance abuse), those with prominent manic symptoms, and those who may experience serious drug interactions (e.g., recent use of antidepressants). Furthermore, only about 35–50% of admitted trial subjects complete the 3-week trials [2,3,4].

One way to reveal the limitations of placebo-controlled (drug wash-out) trials for acute mania is to compare these trials with those that are not placebo controlled. The dose of lorazepam is useful for this assessment since washout trials use sizably different doses of lorazepam for agitation during bipolar mania episodes compared to lorazepam doses used in non-placebo trials and in crisis treatment. Differences in the use of benzodiazepines for agitation in these settings could reveal useful clinical treatment information since the intensity of agitation parallels manic severity [3]. Thus, low levels of agitation in manic episodes suggest a relatively mild degree of bipolar mania. When the degree of drug treatment for manic agitation varies widely by trial methodology, it raises the question of treatment generalizability. Essentially, does adequate drug treatment use for very mild manic cases apply to severe cases in crisis?

## 2 Methods

All 22 of the 3-week, randomized, placebo-controlled drug trials of adults diagnosed with bipolar 1 mania listed in the 2018 review by Morsel et al. [1] were identified and included in the assessment of lorazepam available for use. Nearly all (19 out of 22) recorded the percent of subjects in the drug and placebo arms who were given lorazepam for agitation during the pre-trial and early trial period. Data on the dose of lorazepam/day administered were far less available. Only 8 of the 22 trial reports included that information.

Prior to the placebo-controlled drug trials, all the subjects were taken off existing antimanic medication treatments -- except for lorazepam and hypnotics. This wash-out period lasts for a period usually of 2–7 days. All subjects were hospitalized initially for 2 weeks. During the ‘pre-trial drug washout’ time, the subjects were authorized to receive at most 8 mg of oral lorazepam a day for agitation (usually on day one). During the first 4 days of the trial, lorazepam was available to agitated subjects at doses usually up to 4–6 mg/day. Doses up to 2 mg/day were generally used for the next 6 trial days.

Six trials of non-placebo-controlled, non-wash-out trials of benzodiazepines vs. antimanic drugs were located. These trials evaluated the comparative effectiveness of benzodiazepines to reduce manic symptoms, which included agitation.

For the sake of completeness, I have separately identified 7 complex antimanic drug trials listed in Morsel et al. [1]. They compared treatment outcome of subjects receiving 2 antimanic drugs to subjects receiving one antimanic drug with a placebo. Lorazepam was authorized for both groups as needed for agitation. The trials included inpatients as well as outpatients, and lasted 3–6 weeks. Unfortunately, only one of these reports [5] recorded the doses actually administered.

To identify the doses of benzodiazepine administered orally to patients diagnosed with bipolar mania admitted to hospitals on an emergency basis, I searched for controlled drug trials of patients diagnosed with mania in PubMed, Web of Science, PsycINFO, Google, and Embase. Keywords were clonazepam, agitation, lorazepam and mania. Controlled trials of benzodiazepines for agitation, but not specifically for acute bipolar mania, were excluded, as were case series reports. Fortunately, two overlapping reviews were available [6,7], which included nearly all of the controlled lorazepam/clonazepam trial publications for agitation during acute manic episodes.

## 3 Results

Most subjects diagnosed with bipolar 1 mania who participated in randomized, placebo-controlled, 3-week drug trials were scheduled to receive lorazepam orally if needed for agitation. Pre-trial antimanic drug washout was routine in these trials, so that the trial subjects were treated only with lorazepam as needed. In the 22 trials recorded on Table 1, the rate of oral lorazepam use is listed. A median of 79% of the trial subjects received lorazepam usually before day 10. Drug and placebo subjects received lorazepam at a very similar rate. (Table 1).

**Table 1:** Lorazepam use during placebo-controlled 3-week clinical trials for bipolar mania.

Investigator [reference]	Antimanic Drug	No. of Subjects Drug/placebo	% of Lorazepam Use	
			Antimanic drug	Placebo
Pope et al., 1991 [8]	Valproate	17/19	100	100
Bowden et al., 1994 [9]	Divalproex*	68/73	Not reported	
Tohen et al., 1999 [10]	Olanzapine	70/69	86	86
Tohen et al., 2000^a^ [11]	Olanzapine	54/56	66	73
Keck et al., 2003 [12]	Aripiprazole	131/66	86	85
Keck et al., 2003 [13]	Ziprasidone	130/132	77	79
Hirschfeld et al., 2004 [2]	Risperidone	127/119	81	82
Weisler et al., 2004 [14]	Carbamazepine	101/103	71	67
Weisler et al., 2005 [15]	Carbamazepine	122/117	74	79
Potkin et al., 2005 [16]	Ziprasidone	139/66	73	62
Khanna et al., 2005 [3]	Risperidone	146/144	99	99
Bowden et al., 2006 [17]	Divalproex	192/185	74	79
Sachs et al., 2006 [4]	Aripiprazole	137/135	Not reported	
Keck et al., 2009 [18]	Aripiprazole*	155/165	92	87
Hirschfeld et al., 2010 [19]	Divalproex	147/78	86	86
El Mallakh et al., 2010 [20]	Aripiprazole	131/134	Not reported	
McIntyre et al., 2010 [21]	Asenapine*	185/90	56	60
Cutler et al., 2011 [22]	Quetiapine	149/159	41	49
Kanba et al., 2014 [23]	Aripiprazole	122/125	81	77
Sachs et al., 2015 [24]	Cariprazine	158/154	80	75
Calabrese et al., 2015 [25]	Cariprazine	336/161	63	58
Landbloom et al., 2016 [26]	Asenapine	241/126	55	52

Subjects were taken off antimanic medication for 2–7 days before entering the trial.

a 4-week trial *3 arm trial All subjects were treated as inpatients.

In the 8 trials that listed the administered dose of lorazepam for agitation, the median dose was 2.1 mg/day for the first 7–10 days. Again, the doses were very similar for drug and placebo subjects (Table 2).

**Table 2:** Mean daily dose of lorazepam for drug vs. placebo clinical trials of manic subjects.

Investigator [reference]	Antimanic Drug	Duration of Lorazepam Use	Mean mg/day Dose with Antimanic Drug	Lorazepam Dose with Placebo
Pope et al., 1991 [8]	Valproate	10 days	0.6	1.4
Tohen et al., 1999 [10]	Olanzapine	10 days	1.1	1.7
Tohen et al., 2000 [11]	Olanzapine	10 days	0.6	0.7
Keck et al., 2003 [12]	Ziprasidone	7 days	2.4	3.1
Weisler et al., 2004 [13]	Carbamazepine	7 days	2.2	2.2
Khanna et al., 2005 [3]	Risperidone	10 days	4.1	4.4
Potkin et al., 2005 [15]	Ziprasidone	7 days	2.2	2.0
Bowden et al., 2006 [16]	Divalproex	1 day	2.1	2.4

In non-industry financed controlled trials of benzodiazepines to treat manic episodes, clonazepam was used more than lorazepam. These were 3-week comparative benzodiazepine trials that were not placebo-controlled, and were not preceded by a drug washout. The trial format was to compare a benzodiazepine with an anti-psychotic or lithium with a benzodiazepine alone. The daily benzodiazepine dose used during the first two weeks of the trials ranged from 5.4 to 16 mg a day (Table 3).

**Table 3:** Clonazepam and lorazepam doses used in controlled trials for bipolar mania.

Investigator [reference]	Benzodiazepine treatment	Sample Size	Days of use	Mean daily dose (mg) or dose range (mg/day)
Chouinard et al., 1983 [27]	Clonazepam**	12	10	mean = 10.4
Bradwejn et al., 1990 [28]	Lorazepam	13	7 & 14	mean = 12 & 13
Bradwejn et al., 1990 [28]	Clonazepam	11	7 & 14	mean = 11 & 14
Edwards et al., 1991 [29]	Clonazepam***	20	5	mean = 6
Chouinard et al., 1993 [30]	Clonazepam^##^	8	2 Hours	2-10 range; mean = 5.4
Clark et al., 1997 [31]	Clonazepam*	15	28	8-16 range

The trials were randomized and controlled.

All patients were treated during an inpatient stay.

***Compared to placebo and with chlorpromazine as needed.

##Intramuscular administration and compared to IM haloperidol.

**Compared to lithium in a cross-over design.

*Compared to lithium.

Seven studies from the 2018 Morsel et al. [1] review listed lorazepam usage during trials of concomitant antimanic medications compared to those receiving one antimanic drug plus a placebo. The use of lorazepam for agitation was similar for subjects receiving an antimanic drug plus placebo to those receiving combination antimanic drugs. The average daily dose of lorazepam was not reported. Fewer outpatients were given lorazepam for bipolar manic agitation than inpatients (Table 4).

**Table 4:** Lorazepam use in combination drugs vs antimanic drug + placebo for mania.

Investigator [reference]	Combination of Antimanic Drugs	No. of Weeks	Inpt or Outpt	% of Lorazepam use	
				Combined Drugs	Drug + Placebo
Tohen et al., 2002 [32]	Olanzapine + Val or Li	6	Outpt	29	34
Sacks et al., 2002 [33]	Risperidone + Val or Li	3	Inpt	65	59
Yatham et al., 2003 [34]	Risperidone + Val or Li	3	Inpt	72	63
Vieta et al., 2008 [35]	Aripiprazole + Val or Li	6	Outpt	21	25
Berwaerts et al., 2011 [36]	Paliperidone + Val or Li	6	Outpt	51	51
Sacks et al., 2012 [37]	Risperidone + Val or Li	3	Outpt	27	26
Jahangard et al., 2014 [38]	Allopurinol + Val	4	Inpt.	77	67

Val = valproate Li = lithium carbonate Inpt = inpatient Outpt = outpatient.

Subjects entered the trial on an antimanic drug and received another or a placebo.

There were numerous studies that described benzodiazepine doses used in crisis treatment for acute bipolar mania. The reports from emergency departments listed doses of 16–100 mg/day [[39], [40], [41], [42]].

## 4 Discussion

The major findings from this review are: 1) lorazepam used for agitation in subjects diagnosed with bipolar mania who entered into placebo-controlled, drug wash-out trials were consistently administered in low doses. This suggests that agitation in acute bipolar mania was not a major treatment problem for subjects who had been rapidly deprived of antimanic drugs; 2) the dose of lorazepam to treat agitation in non-placebo, non-wash-out trials was 3-fold higher than the dose used for subjects entering placebo-controlled, drug wash-out trials. This could be accounted for by restrictive subject selection in the placebo-controlled trials.

These subjects were not required to experience major antimanic drug withdrawal before the trial, and understandably were less impaired at baseline. 3) the use of lorazepam for agitation in placebo-controlled trials was very similar between those on antimanic trial medication and those on placebo. This suggests that standard antimanic medication alone was not comparatively more useful to reduce agitation during the first 10 days of these trials and that the degree of impairment of these subjects was relatively minor. 4) the addition of lorazepam for agitation in antimanic medication *combination* trials for outpatients appears to have little effect. However, the treatment data are quite incomplete. The similarity of the placebo and the combination with lorazepam suggests that lorazepam was not a useful treatment addition. 5) clonazepam and lorazepam were administered in high doses (16–100 mg/day) to patients treated for an acute, bipolar manic crisis in emergency departments. This suggests that very substantial benzodiazepine doses are needed to result in symptomatic benefit for patients who are acutely manic and agitated.

In an invited commentary related to a bipolar mania, drug washout trial [43], Kerwin [[44] p.18], made the following comments. “I believe that the participants in this study cannot be typical of real-world patients.” “These must be mildly ill patients … I cannot imagine any of my acutely agitated manic patients who have been admitted to my psychiatric intensive care unit being able to participate in this trial.” Edwards et al. [29], Storosum et al. [45], and Vidiz et al. [46] expressed similar views.

The 2–7 day drug washout before entry into the clinical trial clearly suggests that those entering the trial were not acutely impaired. They were hospitalized during the first 2 weeks of the trial mainly for reasons of caution. During this time, manic subjects were only medicated as needed –for agitation-with lorazepam. If these adults were seriously and acutely impaired, placing them on a placebo would raise serious ethical concerns. Specifically, if effective treatment already exists, it is unethical to create a placebo group that will receive no treatment [47].

The great majority of antimanic medication trials were done overseas. Recruiting these less costly subjects is easier; more potential subjects are drug naïve; and there are less stringent trial regulations [48]. The impact of the trials being overseas on the quality of treatment for mania has yet to be carefully assessed.

The similarity of lorazepam use between drug and placebo subjects early in the trials may be because standard antimanic drugs (e.g., divalproex, lithium, olanzapine) take much longer to reduce behavioral pathology. Specifically, oral lorazepam peaks in the body within 20–30 min [49].

The common use of benzodiazepines for bipolar mania has been criticized by many. In a review on the subject, Otheman et al. [49] wrote that “… their use must be very cautious because of the associated risk of misuse and other possible consequences”. However, emergency department (ED) physicians view lorazepam as an important backup for agitation. In treatment guidelines of an ED workgroup, one major recommendation was: “If the initial dose of antipsychotic is insufficient to control agitation, the addition of a benzodiazepine such as lorazepam is preferred to additional doses of the same antipsychotic or to a second antipsychotic” [ [50], p. 31 ].

In my review of the literature to improve this manuscript and to respond to your suggestions, I added the following paragraphs. I concluded that a restrictive subject selection in these trials is common—either too mild or too at risk. So, I added the 2 paragraphs below.

It is apparent--but rarely reported in the trial literature-that very mild cases of agitation in adults diagnosed with bipolar mania were selectively **identified** from within a much larger patient sample. For example, Cole et al. [51] and Tohen et al. [52] detail high levels of preliminary screening (n = 1461 & n = 3115). Observational acknowledgements of high levels of benzodiazepine treatment of mania, on the other hand, were reported mainly in commentaries and in emergency department reports. (see references [29], [39] and [40]).

Excluding manic trial subjects with problematic comorbidities from entering the trials also serves to reduce many at high risk for symptomatology. These exclusions from the trials were seldom numerically totaled. In the Sachs et al. (2006 [53] trial, for example, subjects were rejected from trial consideration if they met at least one of eleven criteria –including psychotic features, suicidality, a seizure history, alcoholism, and substance abuse.

Sleep loss is a known trigger for bipolar mania [54]. Thus, medications such as clonazepam can aid in inducing sleep [54], and in the process reduce manic agitation [55]. However, the value of benzodiazepine usage beyond the early phase of mania treatment is questionable.

All cause discontinuation rates during the 3-week antimanic controlled trials were variable, but often >35%. Most of the dropout was for lack of effectiveness. Notably, high levels of premature trial discontinuation are not uncommon in adult bipolar subjects generally [56].

Limitations: Details of benzodiazepine use were seldom reported in large, industry-sponsored, controlled antimanic medication trials. Additionally, intramuscular administration of lorazepam is rarely mentioned in the antimanic trials cited.

## 5 Conclusion

An analysis of the low use of lorazepam for agitation by subjects entering placebo-controlled, drug washout trials for bipolar mania suggests that the findings from these trials have limited generalizability for crisis psychiatric practice.

## Declaration of competing interest

The authors declare that they have no known competing financial interests or personal relationships that could have appeared to influence the work reported in this paper.

## References

[1] Morsel A.M., Morrens M., Sabbe B. (2018). An overview of pharmacotherapy for bipolar 1 disorder. Expet Opin. Pharmacother..

[2] Hirschfeld R.M., Keck P.E., Kramer M., Karcher K., Canuso C., Eerdekens M., Grossman F. (2004). Rapid antimanic effect of risperidone monotherapy: a 3-week multicenter, double-blind. Placebo-controlled trial. Am. J. Psychiatr..

[3] Khanna S., Vieta E., Lyons B., Grossman F., Eerdekens M., Kramer M. (2005). Risperidone in the treatment of acute mania. Br. J. Psychiatry.

[4] Sachs G.S., Sanchez R., Marcus R., Stock E., McQuade R., Carson W. (2006). Aripiprazole in the treatment of acute manic or mixed episodes in patients with bipolar 1 disorder: 3-week placebo-controlled study. J. Psychopharmacol..

[5] Machado-Viera R., Soares J.C., Lara D.R., Luckenbaugh D.A., Busnello J.V., Marca G., Cunha A. (2008). A double-blind, randomized, placebo-controlled 4-week study on the efficacy and safety of purinergic agents allopurinol and dipyridamole adjunctive to lithium in acute bipolar mania. J. Clin. Psychiatry.

[6] Bottai T., Hue D., Hillaire-Buys D., Barbe A., R Alric, Pouget R., Petit P. (1995). Clonazepam in acute mania: time-blind evaluation of clinical response and concentrations in plasma. J. Affect. Disord..

[7] Curtin F., Schutz P. (2004). Clonazepam and lorazepam in acute mania: a Bayesian meta-analysis. J. Affect. Disord..

[8] Pope H.G., McElroy S.L., Keck P.E., Hudson J.I. (1991). Valproate in the treatment of acute mania. Arch. Gen. Psychiatr..

[9] Bowden C.L., Brugger A.M., Swann A.C., Calabrese J.R., Janicak P.G., Petty F., Disaver S.C., Davis J.M. (1994). Efficacy of divalproex vs lithium and placebo in the treatment of mania. JAMA.

[10] Tohen M., Sanger T.M., McElroy S.L., Tollefson G.D., Chengappa R., Daniel D.G., Petty F. (1999). Olanzapine versus placebo in the treatment of acute mania. Am. J. Psychiatr..

[11] Tohen M., Jacobs T.G., Grundy S.L., McElroy S.L., Banov M.C., Janicak P.G. (2000). Efficacy of olanzapine in acute bipolar mania. Arch. Gen. Psychiatr..

[12] Keck P.E., Marcus R., Toukodimitris S., Ali M., Liebeskind A., Saha A., Ingenito G. (2003). A placebo-controlled, double-blind study of the efficacy and safety of aripiprazole in patients with acute bipolar mania. Am. J. Psychiatr..

[13] Keck P.E., Versiani M., Potkin S., West S.A., Giller E., Ice K. (2003). Ziprasidone in the treatment of acute bipolar mania: a three-week, placebo-controlled, double-blind, randomized trial. Am. J. Psychiatr..

[14] Weisler R.H., Kalali A.H., Ketter T.A. (2004). A multicenter, randomized, double-blind, placebo-controlled trial of extended-release carbamazepine capsules as monotherapy for bipolar disorder patients with manic or mixed episodes. J. Clin. Psychiatry.

[15] Weisler R.H., Keck P.E., Swann A.C., Cutler A.J., Ketter T.A., Kalali A.H. (2005). Extended-release carbamazepine capsules as monotherapy for acute mania in bipolar disorder a multicenter, randomized, double-blind, placebo-controlled trial. J. Clin. Psychiatry.

[16] Potkin S.G., Keck P.E., S Segal K Ice, English P. (2005). Ziprasidone in acute bipolar mania. J. Clin. Psychopharmacol..

[17] Bowden C.L., Swann A.C., Calabrese J.R., Rubenfaer L.M., Wozniak P.J., Collins M.A., Abi-Saab W., Saltarelli M. (2006). A randomized, placebo-controlled, multicenter study of divalproex sodium extended release in the treatment of acute mania. J. Clin. Psychiatry.

[18] Keck P.E., Orsulak P.J., Cutler A.J., Sanchez R., Torbeyns A., Marcus R.N., McQuade R.D., Carson W.H. (2009). Aripiprazole monotherapy in the treatment of acute bipolar 1 mania: a randomized, double-blind, placebo- and lithium-controlled study. J. Affect Discord..

[19] Hirschfeld R.M., Bowden C.L., Vigna N.V., Wozniak R., Collins M. (2010). A randomized, placebo-controlled, multicenter study of divalproex sodium extended-release in the acute treatment of mania. J. Clin. Psychiatry.

[20] El Mallakh R.S., E Vieta L Rollin, Marcus R., Carson W.H., McQuade R. (2010). A comparison of two fixed doses of aripiprazole with placebo in acutely relapse, hospitalized patients with bipolar 1 (manic or mixed) in subpopulations. Eur. Neuropsychopharmacol.

[21] McIntyre R.S., Cohen M., Zhao J., Alphs L., Macek T.A., Panagides J. (2010). Asenapine in the treatment of acute mania in bipolar 1 disorder: a randomized, double-blind, placebo-controlled trial. J. Affect. Disord..

[22] Cutler A.J., Datto C., Nordenhem A., Minkwitz M., Acevedo L., Darko D. (2011). Extended-release quetiapine as monotherapy for the treatment of adults with acute mania: a randomized, double -blind, 3-week trial. Clin. Therapeut..

[23] Kamba S., Kawasaki H., Ishigooka J., Sakamoto K., Kinoshita T., Kuroki T. (2014). A placebo-controlled, double-blind study of the efficacy and safety of aripiprazole for the treatment of acute manic or mixed episodes in Asian patients with bipolar 1 disorder (the AMAZE study). World J. Biol. Psychiatr..

[24] Sachs G.S., Greenberg W.M., Starace A., Lu K., Rut A., Laszlovszky I., Nemeth G., Durgam S. (2015). Cariprazine in the treatment of acute mania in bipolar 1 disorder: a double-blind, placebo-controlled, phase 111 trial. J. Affect. Disord..

[25] Calabrese J.R., Keck P.E., Starace A., Lu K., Ruth A., Istvan-Laszlovszky I., Nemeth G., Durgam S. (2015). Efficacy and safety of low-and high-dose cariprazine in acute and mixed mania associated with bipolar 1 disorder: a double-blind, placebo-controlled study. J. Clin. Psychiatry.

[26] Landbloom R.L., Mackle M., Wu X., Kelly L., Snow-Adami L., McIntyre R.S., Mathews M., Hundt C. (2016). Asenapine: efficacy and safety of 5 and 10 mg bid in a 3-week, randomized, double-blind, placebo-controlled trial of adults with a manic or mixed episode associated with bipolar 1 disorder. J. Affect. Disord..

[27] Clouinard G., Young S.N., Annable L. (1983). Antimanic effect of clonazepam. Biol. Psychiatr..

[28] Bradwejn J., Shriqui C., Koszycki D., Meterissian G. (1990). Double-blind comparison of the effects of clonazepam and lorazepam in acute mania. J. Clin. Psychopharmacol..

[29] Edwards R., Stephenson U., Flewett T. (1991). Clonazepam in acute mania; a double-blind trial. Aust. NZ J. Psychiatr..

[30] Chouinard G., Annable L., Turnier L., Holobow N., Szkrumelak N. (1993). A double-blind randomized clinical trial of rapid tranquilization with I.M. clonazepam and I.M. haloperidol in agitated psychotic patients with manic symptoms. Can. J. Psychiatr..

[31] Clark H.M., Berk M., Brook S. (1997). A randomized controlled single blind study of the efficacy of clonazepam and lithium in the treatment of acute mania. Hum. Psychopharmacol..

[32] Tohen M., Chengappa K.N., Suppes T., Zarate C.A., Calabrese J.R., Bowden C.L., Sachs G.S. (2002). Efficacy of olanzapine in combination with valproate or lithium in the treatment of mania in patients partially nonresponsive to valproate or lithium monotherapy. Arch. Gen. Psychiatr..

[33] Sachs G.S., Grossman F., Ghaemi S.N., Okamoto A., Bowden C.L. (2002). Combination of a mood stabilizer with risperidone or haloperidol for treatment of acute mania: a double-blind, placebo-controlled comparison of efficacy and safety. Am. J. Psychiatr..

[34] Yatham L.N., Grossman F., Augustyns I., Vieta E., Ravidran A. (2003). Mood stabilizers plus risperidone or placebo in the treatment of acute mania. Br. J. Psychiatry.

[35] Vieta E., Tjoen C., McQuade R.D., Carson W.H., Marcus R.M., Sanchez R., Owen R., Nameche L. (2008). Efficacy of adjunctive aripiprazole to either valproate or lithium in bipolar mania patients partially nonresponsive to valproate/lithium monotherapy; a placebo-controlled study. Am. J. Psychiatr..

[36] Berwaerts J., Lane R., Nuamah I.F., Lim P., Remmerie B., Hough D.W. (2011). Paliperidone extended-release as adjunctive therapy to lithium or valproate in the treatment of acute mania: a randomized, placebo-controlled study. J. Affect. Disord..

[37] Sachs G.S., Vanderburg D.G., Karayal O.N., Kolluri S., Bachinsky M., Cavus I. (2012). Adjuctive oral ziprasidone in patients with acute mania treated with lithium or divalproex, part 1: results of randomized, double-blind, placebo-controlled trial. J. Clin. Psychiatry.

[38] Jahangard L., Soroush S., Haghighi M., Ghaleiha A., Bajoghli H., Holsboer-Trachsler E., Brand S. (2014). In a double-blind, randomized and placebo-controlled trial, adjuvant allopurinol improved symptoms of mania in in-patients suffering from bipolar disorder. Eur. Neuropsychopharmacol.

[39] Chouinard G. (1985). Antimanic effects of clonazepam. Psychosomatics.

[40] Lenox R.H., Modell J.G., Weiner S. (1986). Acute treatment of manic agitation with lorazepam. Psychosomatics.

[41] Rakofsky J.J., Dunlop B.W. (2014). Treating bipolar mania in the outpatient setting: risk and reward. Curr. Psychiat..

[42] Santos A.B., Morton W.A. (1989). Use of benzodiazepines to improve management of manic agitation. Hosp. Community Psychiatr..

[43] Meehan K., Zhang F., David S., Tohen M., Janicak P., Small J. (2001). A double-blind, randomized comparison of the efficacy and safety or intramuscular injections of olanzapine, lorazepam. or placebo in treating acutely agitated patients diagnosed with bipolar mania. J. Clin. Psychopharmacol..

[44] Kerwin R. (2002). Olanzapine was more effective than lorazepam at 2 hours but not at 24 hours in bipolar mania with acute agitation. Evid-based MH.

[45] Storosum J.G., Fouwels A., Gispen de Wied C., Wohlfarth T., van Zwiten B., van den Brink W. (2004). How real are patients in placebo-controlled studies of acute manic episode?. Eur. Neuropsychopharmacol.

[46] Yidiz A., Guleryuz S., Ankerst D.P., Olgur D., Renshaw P.F. (2008). Protein kinase C inhibition in the treatment of mania: a double-blind, placebo-controlled trial of tamoxifen. Arch. Gen. Psychiatr..

[47] Gupta U., Verma M. (2013). Placebo in clinical trials. Perspect Clin. Res..

[48] Hull D. (2015). Reining in the commercialized foreign clinical trial. J. Leg. Med..

[49] Otheman Y., Kadirl M., Mehsanni J., Bichra M.Z. (2018). The use of benzodiazepines in bipolar disorders. Addict. Clin. Res..

[50] Wilson M.P., Pepper D., Currier G.W., Holloman G.H., Feifel D. (2012). The psychopharmacology of agitation: consensus statement of the American association of emergency project BETA psychopharmacology workgroup. West. J. Emerg. Med..

[51] Cole J.B., Kline L.R., Millinax S.Z., Nordstrom K.D., Driver B.E., Wilson M.P. (2019). Study enrollment when “preconsent” is utilized for a randomized clinical trial of two treatments for acute agitation in the emergency department. Acad. Emerg. Med..

[52] Tohen M., Vieta E., Pinto A.G., Reed C., Lin D. (2010). Baseline characteristics and outcomes in patients with first episode or multiple episodes of acute mania. J. Clin. Psychiatry.

[53] Sachs G., Sanchez R., Marcus R., Stock E., McQuade R., Carson W. (2006). Aripiprazole in the treatment of acute manic or mixed episodes in patients with bipolar 1 disorder: a 3-week placebo-controlled study. J. Psychopharmacol..

[54] Lewis K.S., Gordon-Smith K., Forty L., Di Florio A., Craddock N., Jones L., Jones I. (2017). Sleep loss as a trigger of mood episodes in bipolar disorder: individual differences based on diagnostic subtype and gender. Br. J. Psychiatry.

[55] Minkel J., Krystal A.D. (2013). Optimizing the pharmacologic treatment of insomnia: current status and future horizons. Sleep Med. Clin..

[56] Kishi T., Sakuma K., Okuya M., Matsuda Y., Esumi S., Hashimoto Y., Hatano M., Miyake N. (2020). Recurrence of mania or depression among adult bipolar patients who continued using lithium. J. Clin. Psychopharmacol..

